# Altered processing enhances the efficacy of small-diameter silk fibroin vascular grafts

**DOI:** 10.1038/s41598-019-53972-y

**Published:** 2019-11-25

**Authors:** Alex H. P. Chan, Elysse C. Filipe, Richard P. Tan, Miguel Santos, Nianji Yang, Juichien Hung, Jieyao Feng, Sidra Nazir, Alexander J. Benn, Martin K. C. Ng, Jelena Rnjak-Kovacina, Steven G. Wise

**Affiliations:** 1 The Heart Research Institute, 7 Eliza Street, Newtown, Sydney, NSW 2042 Australia; 2 Sydney Medical School, University of Sydney, Sydney, NSW 2006 Australia; 3 Garvan Institute of Medical Research & The Kinghorn Cancer Center, Sydney, NSW 2010 Australia; 4 St Vincent’s Clinical School, Faculty of Medicine, UNSW Sydney, Sydney, NSW 2010 Australia; 5 Department of Cardiology, Royal Prince Alfred Hospital, Camperdown, Sydney, NSW 2050 Australia; 6 Graduate School of Biomedical Engineering, UNSW Sydney, Sydney, NSW 2052 Australia; 7 School of Medical Sciences, Dept of Physiology, University of Sydney, Sydney, NSW 2006 Australia; 8 Charles Perkins Centre, University of Sydney, Sydney, NSW 2006 Australia

**Keywords:** Biomedical materials, Implants

## Abstract

Current synthetic vascular grafts are not suitable for use in low-diameter applications. Silk fibroin is a promising natural graft material which may be an effective alternative. In this study, we compared two electrospun silk grafts with different manufacturing processes, using either water or hexafluoroisopropanol (HFIP) as solvent. This resulted in markedly different Young’s modulus, ultimate tensile strength and burst pressure, with HFIP spun grafts observed to have thicker fibres, and greater stiffness and strength relative to water spun. Assessment in a rat abdominal aorta grafting model showed significantly faster endothelialisation of the HFIP spun graft relative to water spun. Neointimal hyperplasia in the HFIP graft also stabilised significantly earlier, correlated with an earlier SMC phenotype switch from synthetic to contractile, increasing extracellular matrix protein density. An initial examination of the macrophage response showed that HFIP spun conduits promoted an anti-inflammatory M2 phenotype at early timepoints while reducing the pro-inflammatory M1 phenotype relative to water spun grafts. These observations demonstrate the important role of the manufacturing process and physical graft properties in determining the physiological response. Our study is the first to comprehensively study these differences for silk in a long-term rodent model.

## Introduction

Synthetic vascular grafts made from expanded polytetrafluorethylene (ePTFE) and polyethylene terephthalate (PET) are effective materials for large diameter grafting applications, such as aortic arch replacement^1^. However, for low diameter (<6 mm) applications including coronary artery and peripheral artery bypass, these materials uniformly fail^2^. Both ePTFE and PET present highly hydrophobic, foreign surfaces which increase acute blood clot formation and drive a chronic inflammatory response^3^. The regrowth of the protective endothelial cell layer lining native vessels is significantly delayed on these materials prolonging the risk of adverse events^4^. These permanent polymer conduits allow only limited, if any, cell ingrowth and are not remodelled by the host. The poor performance of synthetic polymers in these applications, and the lack of newly commercialised materials leaves an urgent unmet need for new candidates. Recent advances in purification and materials engineering techniques has made the production of robust vascular conduits from natural materials an exciting alternative.

A promising natural material for biomaterial applications is silk fibroin (referred to as ‘silk’), commonly sourced from *Bombyx mori* cocoons. Silk can be readily engineered into a diverse range of architectures including hydrogels, films, porous scaffolds and micro-/nano- particles^5^. Silk has particular promise as a vascular graft material due to its tuneable physical properties, low inflammatory profile^6^ and compatibility with blood^7^. As a natural, degradable material, silk can be engineered to promote cell infiltration and tissue remodelling. While several methods of graft generation are compatible with silk, electrospinning has some intrinsic advantages, with multiple studies demonstrating modulation of fibre diameter^8^, mechanical properties^9,10^ and blending with other synthetic polymers or proteins to obtain hybrid scaffolds^11,12^. Following several studies exploring the feasibility of making durable synthetic silk conduits, recent advances in silk processing have made mechanically robust conduits feasible for the first time^13^. While physical scaffold parameters such as elasticity, fibre diameter and porosity are known to influence cell infiltration, morphology and signalling in flat scaffolds, the effect of these parameters on long-term graft performance is less clear. Further insight into the role of physical graft parameters is critical if silk conduits are to progress further into pre-clinical evaluation.

In this study, we demonstrate that changing the silk manufacturing process and therefore altering the fibre thickness and porosity of electrospun silk vascular grafts has a profound effect on vascular remodelling in a rat abdominal aorta grafting model. Compared to a water spun (WS) silk graft with relatively low porosity previously developed in our lab^13^, we observed significantly more rapid re-endothelisation on a HFIP spun (HS) silk graft spun with increased fibre diameter and higher porosity. Maturation of neointimal hyperplasia was also accelerated, plateauing at earlier timepoints compared to low porosity grafts and characterised by enhanced deposition of extracellular matrix proteins. An initial examination of early inflammatory events indicated that HS grafts increased the proportion of anti-inflammatory CD206^+^ M2 phenotype macrophages compared to WS grafts. Overall, this work demonstrates that modulating electrospinning parameters to increase fibre and pore size of silk vascular grafts enhances their *in vivo* remodelling by the host, resulting in improved functional outcomes. These findings prompt consideration of other parameters within the manufacturing processes of silk which may further enhance their performance as synthetic vascular grafts.

## Results

### Graft characterisation

#### Physical properties

We aimed to electrospin two silk conduits with significantly different fibre diameters and corresponding porosity. To achieve this, we systematically explored spinning conditions for scaffolds spun from water (Supplementary Fig. 1) and from HFIP (Supplementary Fig. 2). Using water as a solvent was examined using increasing ratios of polyethylene oxide (PEO)/Silk. Solution viscosity trended linearly with PEO concentration, increasing from 0.29 ± 0.01 Pa.s at 2mL silk:1g PEO, up to 1.21 ± 0.03 Pa.s at 2 mL silk:4g PEO (Supplementary Fig. 1A) Quantification of fiber thickness distribution showed that neither flow rate, nor viscosity significantly changed fiber thickness when silk is electrospun from water (Supplementary Fig. 1B) with an average fiber diameter of 355 ± 5 nm for the 2 mL Silk:1g PEO group, not significantly different from any of the other conditions. The greatest variability was seen for 2 mL Silk:3g PEO (Supplementary Fig. 1C). Lyophilized silk was alternatively dissolved in HFIP at concentrations of 5, 10, 15 and 20% and subsequently electrospun at 1.0, 1.5, 2.0 and 2.5 mL/hr. Increasing silk concentration significantly increased solution viscosity (Supplementary Fig. 2A). Electrospinning from 20% silk was not possible as the viscosity was too high to permit fiber formation. Quantification of fiber diameter at 10 or 15% silk showed that higher flow rates resulted in larger average diameter but also a larger distribution (Supplementary Fig. 2B). At 15%, average fiber size increased from 2077 ± 38 nm (1 mL/hr) to 3585 ± 74 nm (2.5 mL/hr). Spinning efficiency for 15% silk at 2.5 mL/hr was low, with much of the solution lost before reaching the collector, making this condition undesirable for further investigation (Supplementary Fig. 2C, asterisk).

**Figure 1:**
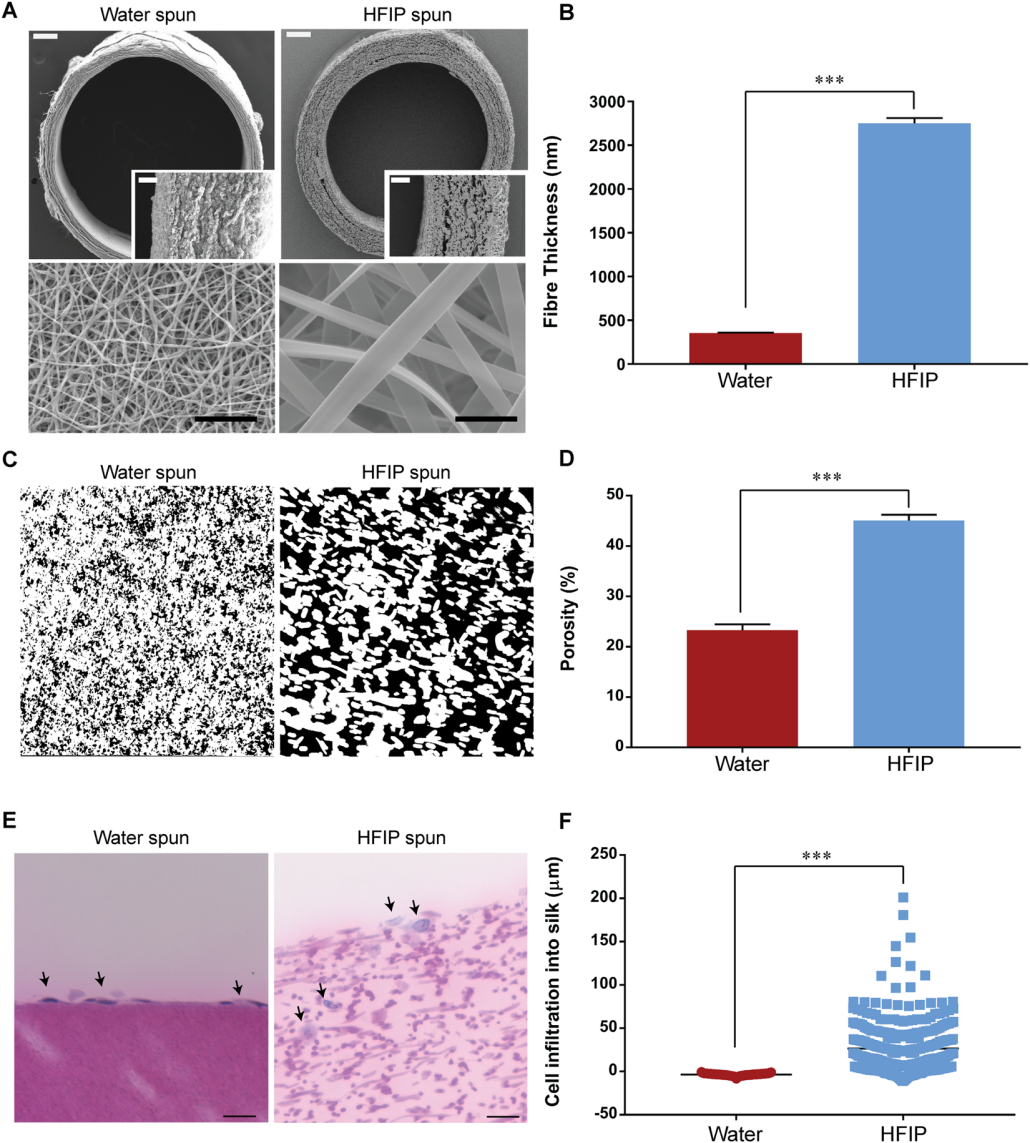
Morphological characterisation of electrospun silk grafts. (**A**) Scanning electron microscopy of electrospun silk grafts. Top row: Cross section of electrospun silk graft. Scale bar = 200 μm, inset scale bar = 50 μm. Bottom row: Luminal surface. Scale bar = 10 μm. (**B**) Quantification of mean fibre thickness, data expressed as mean ± SEM, n = 250 fibres from 6 fields of view. (**C**) Cross section of silk grafts to demonstrate porosity, black indicating space between fibres. (**D**) Quantification of porosity represented as a percentage of the null space, data expressed as mean ± SEM, n = 3. (**E**) Representative image of H&E stained cross sections of endothelial cell seeded scaffolds for 9 days. Black arrows indicate nuclei of cells, scale bar = 50 µm. (**F**) Quantification of cellular infiltration into the silk scaffold represented as depth of nuclei from the surface of scaffold, data expressed as mean ± SEM, n = 3.

From these, an optimal WS graft (2 mL:1 g, 0.7 ml/hr) and HS graft (15% silk, 2.0 ml/hr), containing an overall similar amount of silk, were generated based on conditions best producing low and high fibre diameters. The WS graft consisted of significantly lower fibre diameter compared to HS graft (355 ± 5 nm vs 2750 ± 61 nm) and a reduced wall thickness (264.62 ± 3.0 µM vs 486.99 ± 6.0 µM) (Fig. 1A,B). The relative porosity was also significantly different with the HS graft having a higher porosity compared to the water spun graft (45.1 ± 1.2 % vs 23.3 ± 1.7 %) (Fig. 1C,D). Interconnectivity of the pores was demonstrated by human coronary endothelial cells seeded on HS grafts which infiltrated to a mean depth of 26.67 ± 1.6 µm but formed a monolayer on top of WS graft (Fig. 1E,F). Fourier transform infrared spectroscopy (FTIR) showed β-sheet crystallinity was not significantly different between the two silk grafts (Supplementary Fig. 3A,B). The effective removal of HFIP from scaffolds spun from this solvent was also confirmed using this technique (Supplementary Fig. 3C).

#### Mechanical properties

**Figure 2:**
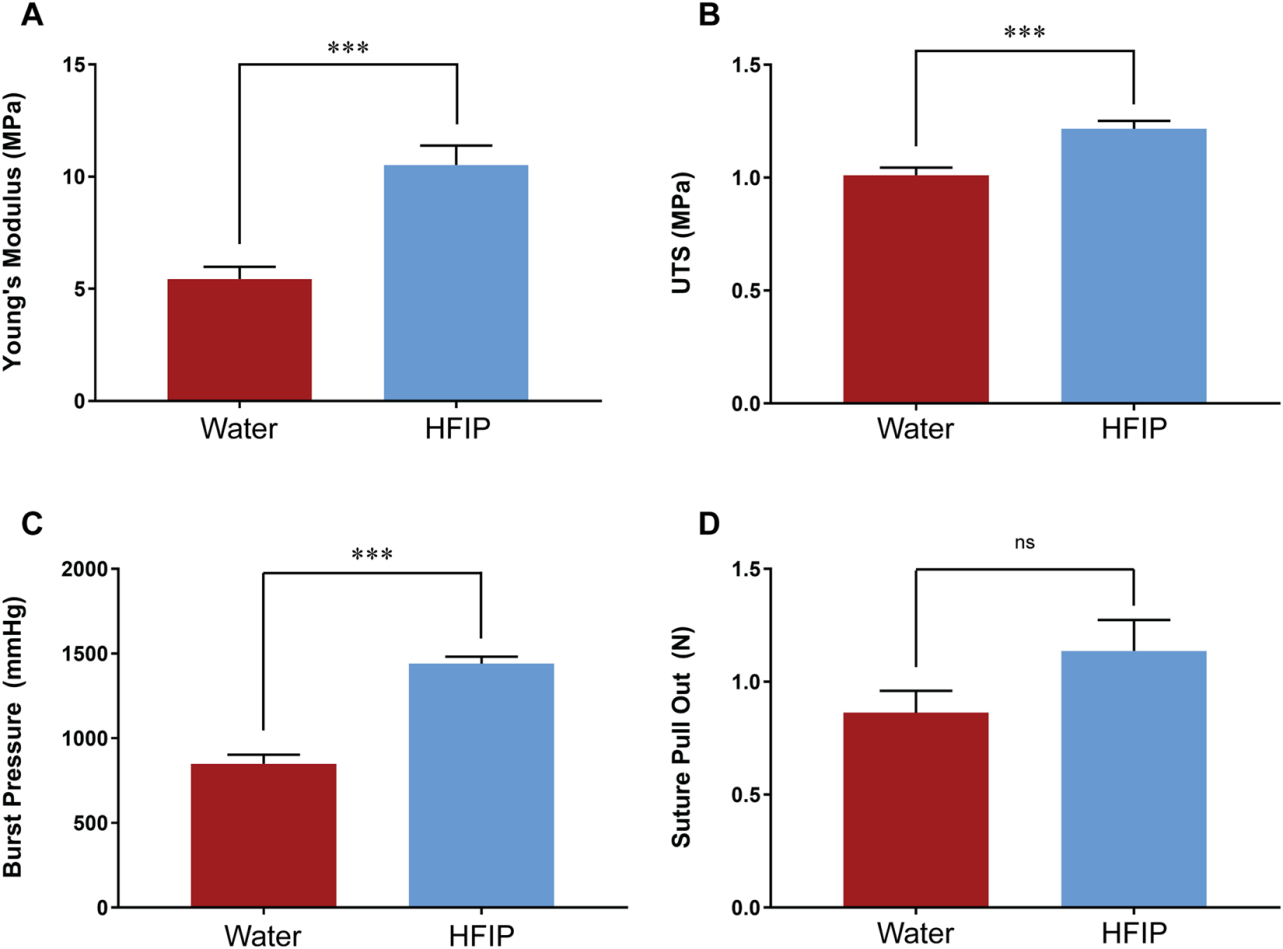
Mechanical properties of electrospun silk grafts. (**A**) Young’s modulus. (**B**) Ultimate tensile strength. (**C**) Maximal burst pressure before failure. (**D**) Suture retention strength represented as the maximal force before failure. Data expressed as mean ± SEM and analysed by t-test, n = 6–9. Data for the WS group were modified from a previous study^13^.

Both HS and WS grafts were water annealed after electrospinning for stabilisation. We studied annealing temperatures of 4, 24, 37 and 55 °C but did not observe a significant effect of this parameter on mechanical properties (Supplementary Fig. 4). Both silk conduits were stiffer than rat aorta controls but significantly more elastic that ePTFE. At 24 °C annealing, HS grafts exhibited a 93 % increase in Young’s modulus relative to WS grafts (10.52 ± 0.9 MPa vs 5.44 ± 0.6 MPa) (Fig. 2A). HS grafts also had 21 % higher ultimate tensile strength (UTS) than WS grafts (1.22 ± 0.03 MPa vs 1.01 ± 0.03 MPa) (Fig. 2B). Burst pressure was 70 % higher in the HS grafts (1441 ± 41 mmHg) compared to WS grafts (849 ± 54 mmHg) (Fig. 2C). Suture retention was not significantly different between two groups (Fig. 2D).

### *In vivo* characterisation

#### Endothelialisation

**Figure 3:**
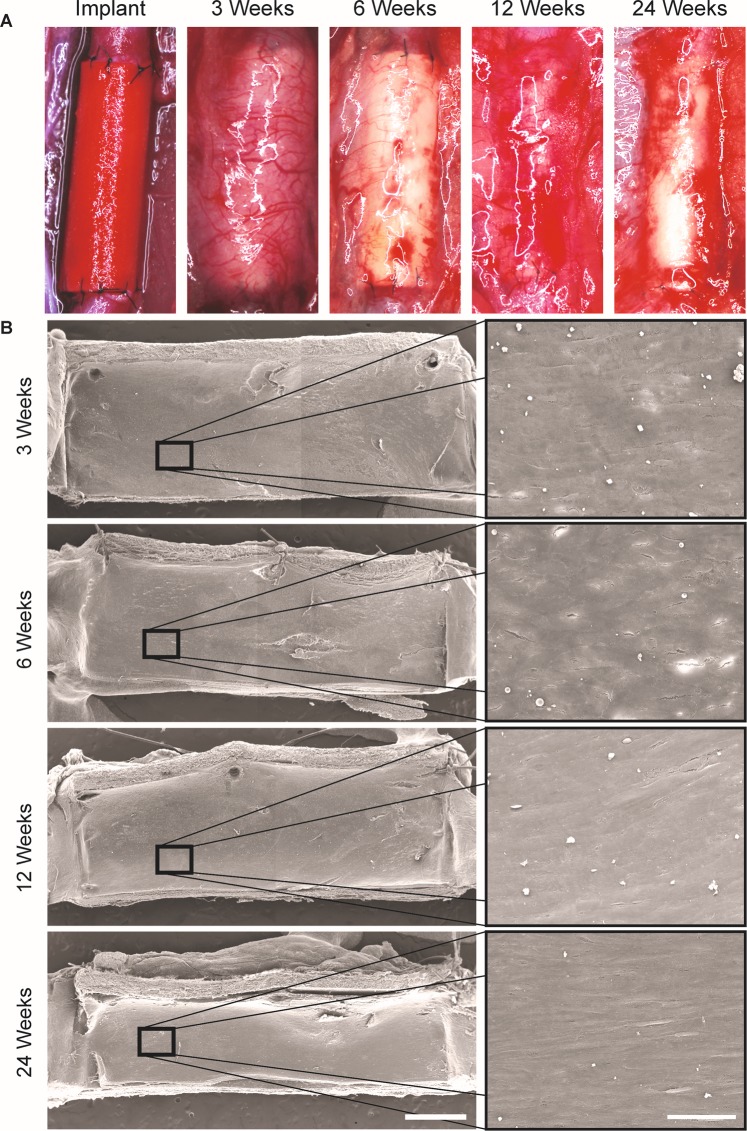
Macroscopic characterisation of electrospun HS silk grafts. (**A**) HS silk grafts at implant and explant timepoints. (**B**) Scanning electron microscopy of luminal surface of explanted grafts. Scale bar = 1 mm, inset scale bar = 50 µm.

**Figure 4:**
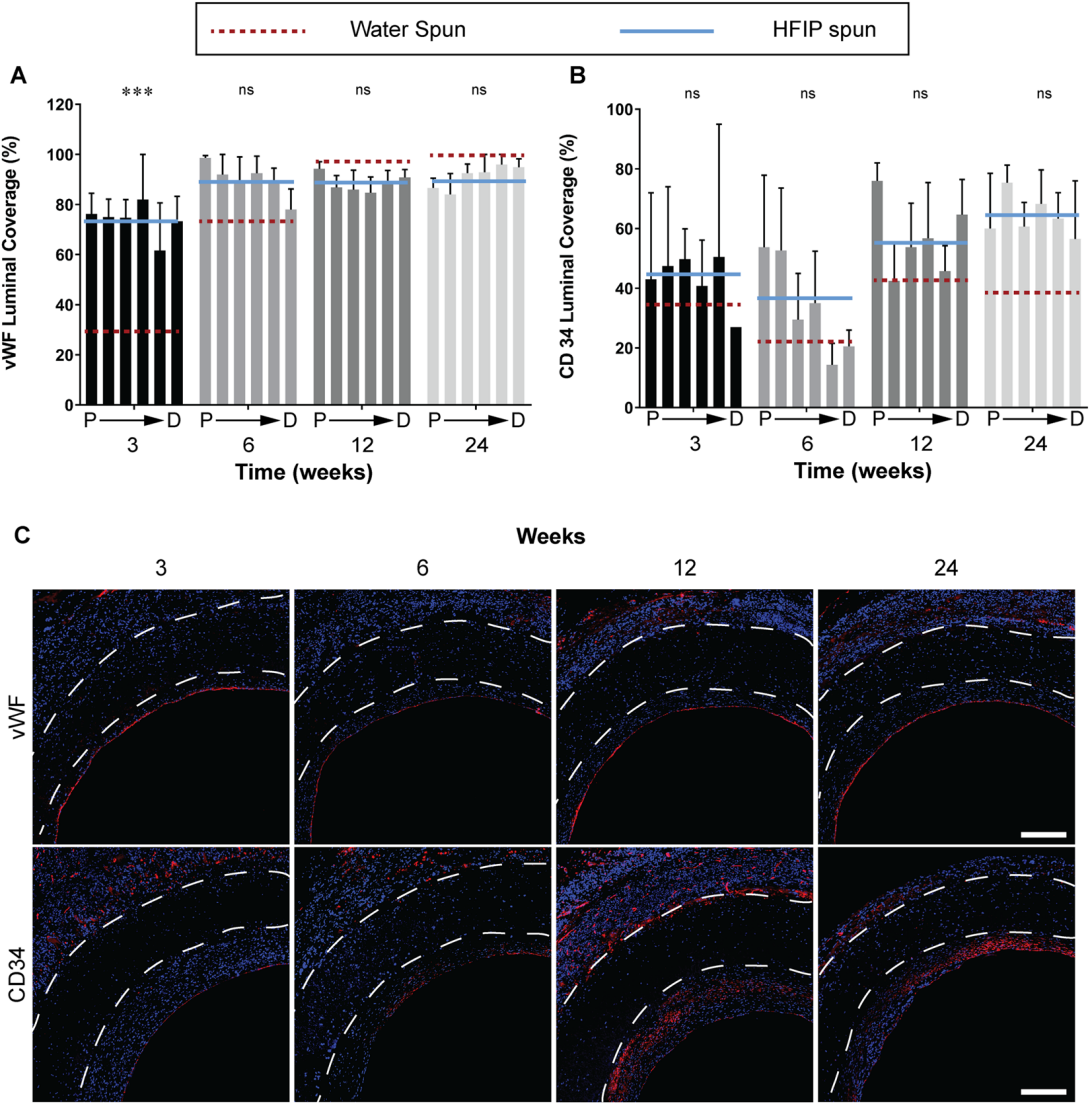
Endothelialisation of silk vascular grafts. Data expressed as mean ± SEM, n = 4 animals/timepoint. The solid blue line represents the average of the bars presented (HFIP spun) and is compared to red dotted line which represents the average of water spun grafts (bars not shown). Statistical analysis comparing these averages was by two-way ANOVA, using Šídák multiple comparison test, with *p < 0.05, **p < 0.01, ***p < 0.001. P → D represents sections of the graft from proximal to distal. (**A**) Quantification of vWF coverage represented as a percentage of total luminal circumference. (**B**) Quantification of CD34 coverage represented as a percentage of total luminal circumference. (**C**) Representative images of cross sections at the mid graft of HS silk vascular grafts, white dotted lines indicate the graft wall. Top row: vWF stained in red and nuclei in blue. Bottom row: CD34 stained in red and nuclei in blue. Scale bar = 200 μm. Data for the WS group were modified from a previous study^13^.

HS grafts were implanted in a rat abdominal aorta grafting model. At explant, all samples were intact and free from aneurysms (Fig. 3A). Scanning electron microscopy was utilised to assess the cellular coverage on the luminal wall of explanted grafts at 3, 6, 12 and 24 weeks. We observed complete coverage, with no exposed luminal electrospun fibres from week 3 post implantation (Fig. 3B). The underlying fibrous structure was visible at the edges of the cross-sections, but high-resolution imaging of the underlying fibers was not possible with the samples available to us. Endothelial cells were identified using immunostaining for vWF, growing on top of the neointimal layer. At week 3 post implantation, we observed a significant 149 % increase in vWF coverage on HS grafts compared to WS grafts (73.7 ± 6.5 % vs 29.6 ± 7.2 %) (Fig. 4A). After week 6 post-implantation, HS grafts showed a trend for an endothelialisation advantage over WS grafts, with near complete endothelialisation at later timepoints. Representative images show a monolayer of vWF^+^ cells at the luminal surface of HS grafts (Fig. 4C, top row). A direct comparison of vWF staining images for WS and HS grafts can be seen in Supplementary Fig. 5.

The contribution of endothelial progenitor cells to graft endothelialisation was assessed through quantification of CD34^+^ cells. We found that the contribution to the endothelium was similar between the two grafts at 3, 6 and 12 weeks, however at 24 weeks, HS grafts contained 69 % higher CD34 luminal coverage with 64.7 ± 4.5 % compared to 38.3 ± 7.4 % for WS grafts. At later timepoints, CD34^+^ cells were found in the neointima as well as the luminal surface of HS grafts (Fig. 4C, bottom row).

#### Neointimal hyperplasia

**Figure 5:**
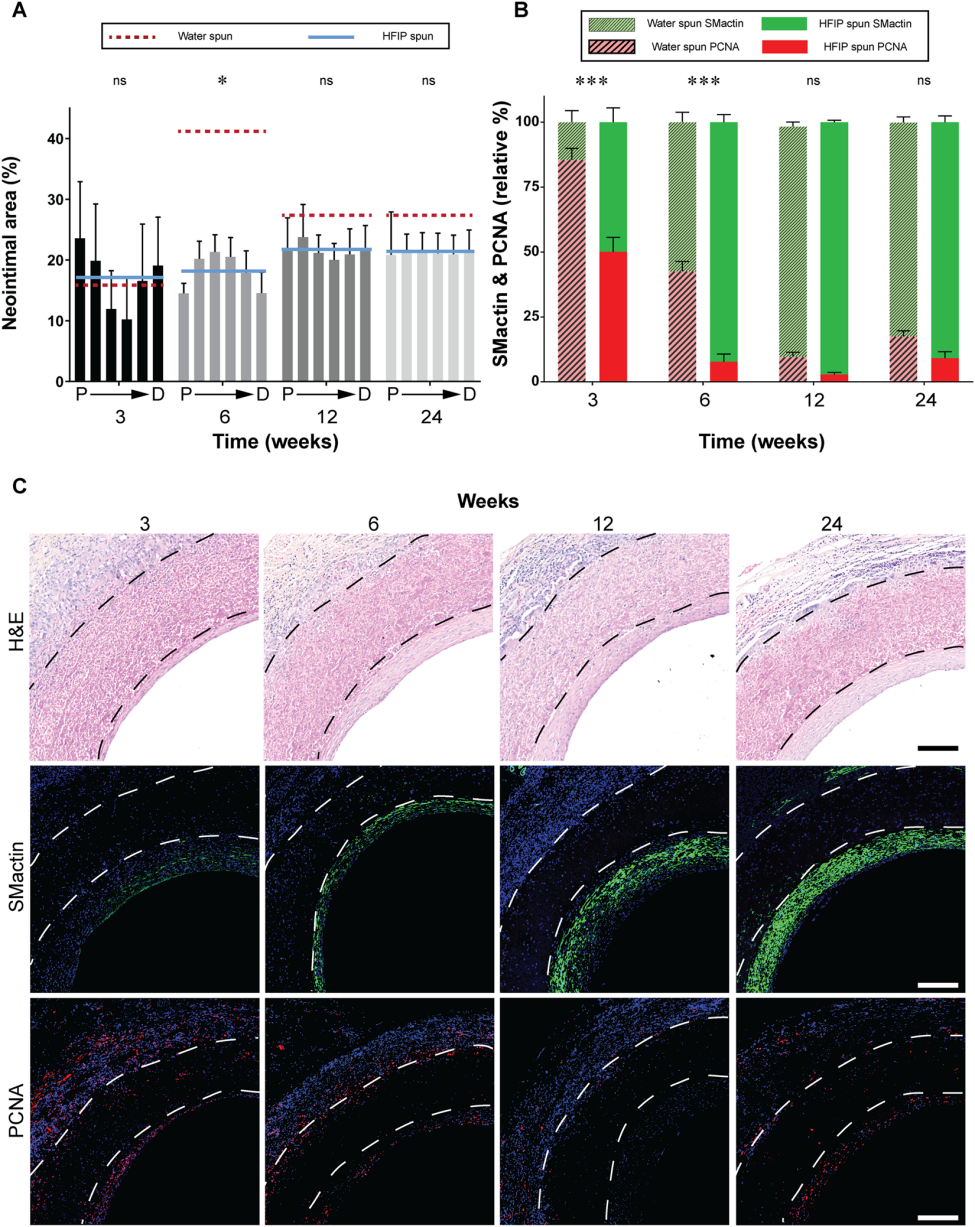
Characterisation of neointimal hyperplasia development. Data expressed as mean ± SEM, n = 4 animals/timepoint. (**A**) Quantification of neointimal area expressed as a percentage of total luminal area defined by the lumen graft wall. The solid blue line represents the average of the bars presented (HFIP spun) and is compared to red dotted line which represents the average of water spun grafts (bars not shown). Statistical analysis comparing these averages was by two-way ANOVA, using Šídák multiple comparison test, with *p < 0.05, **p < 0.01, ***p < 0.001. (**B**) Quantification of SMactin staining and PCNA staining represented as a percentage of total staining in the neointimal area. (**C**) Representative images of cross sections at the proximal end of HS silk vascular grafts, black or white dotted lines indicate the graft wall. Top row: Haematoxylin and eosin staining. Middle row: SMactin stained in green and nuclei in blue. Bottom row: PCNA stained in red and nuclei in blue. Scale bar = 200 μm. Data for the WS group were modified from a previous study^13^.

H&E staining showed the graft walls were largely preserved with only gradual degradation and cell infiltration over this time course. The neointimal area of HS silk increased gradually from 16.9 ± 7.9 % at week 3, before plateauing at 21.6 ± 2.3 % at week 12. This contrasts with WS grafts where a sharp increase in neointimal area up to 40.9 ± 8.4 % was observed at week 6, 124 % higher compared to HS grafts, before a regression back to 27.0 ± 2.1 % at week 24 (Fig. 5A). At week 3, neointimal area was greatest at the graft anastomoses for HS grafts, increasing in uniformity over time (Fig. 5A). Representative images of H&E stained cross sections of HS grafts show a steady increase over time.

Contractile and synthetic SMC phenotypes were assessed using SMactin and PCNA, respectively, to determine SMC composition in the neointima. Neointima possessing a higher SMactin relative to PCNA indicates increased stability and less cell proliferation. HS grafts showed a more rapid maturation of the neointima, indicated by reduced relative PCNA staining at week 3 and 6 post-implantation, when compared to WS grafts (7.8 ± 1.6 % vs 42.6 ± 2.1 %) (Fig. 5B). At longer timepoints of week 12 and 24, HS grafts maintained higher levels of SM actin relative to WS grafts, but these differences were less pronounced compared to earlier timepoints (Fig. 5B). This was reflected clearly in the representative immunostaining images, with increase in SM actin stained in green (Fig. 5C, middle row) and decrease in proliferating cells stained in red (Fig. 5C, bottom row). A direct comparison of H&E, SM actin and PCNA stained images for WS and HS grafts can be seen in Supplementary Fig. 6.

#### Extracellular matrix deposition

**Figure 6:**
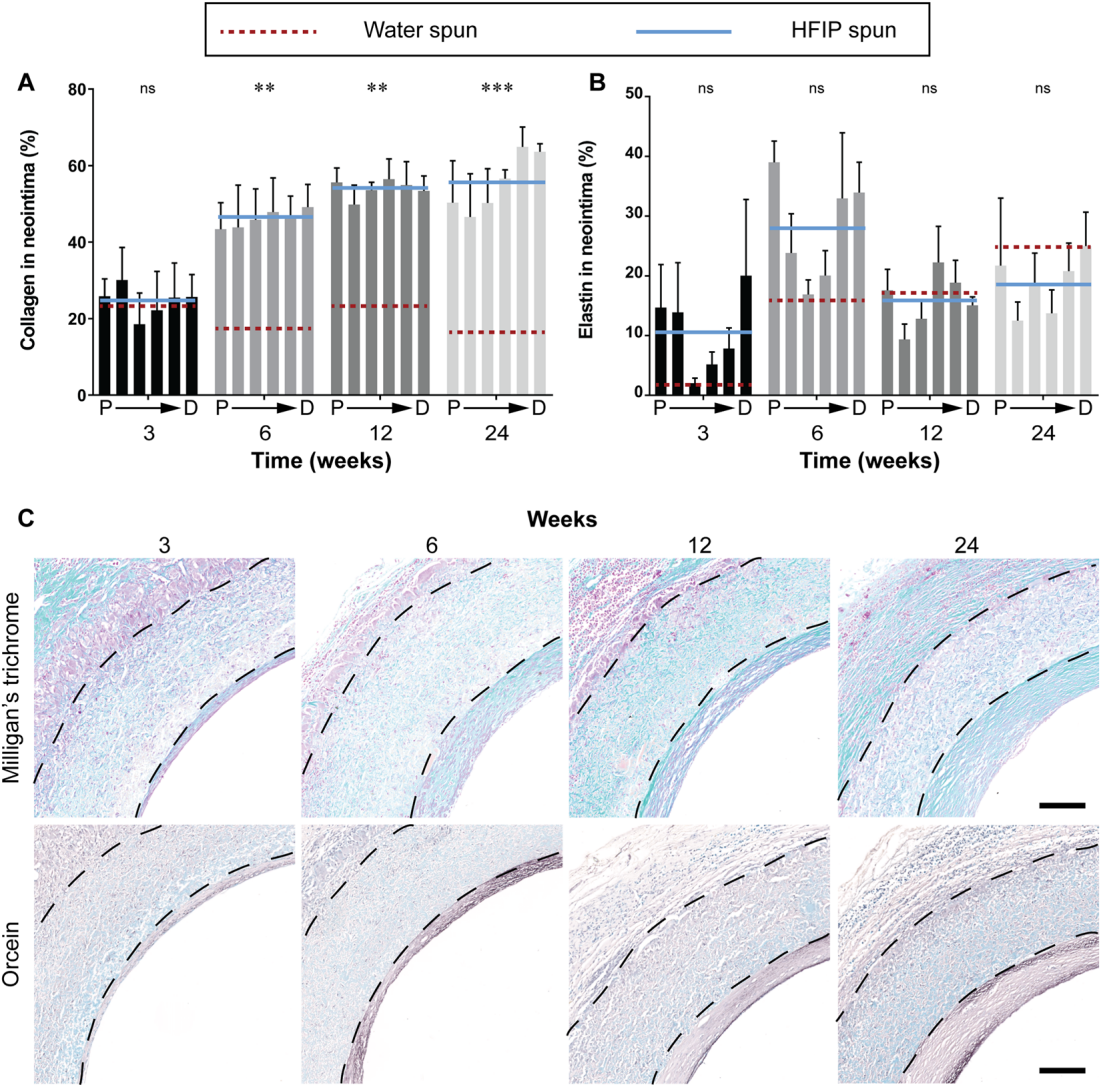
Extracellular matrix deposition in the neointima. Data expressed as mean ± SEM, n = 4 animals/timepoint. The solid blue line represents the average of the bars presented (HFIP spun) and is compared to red dotted line which represents the average of water spun grafts (bars not shown). Statistical analysis comparing these averages was by two-way ANOVA, using Šídák multiple comparison test, with *p < 0.05, **p < 0.01, ***p < 0.001. P → D represents sections of the graft from proximal to distal. (**A**) Quantification of total collagen in the neointima represented as a percentage of the neointimal area. (**B**) Quantification of elastin in the neointima represented as a percentage of the neointimal area. (**C**) Representative images of cross sections at the proximal end of HS silk vascular grafts, black dotted lines indicate the graft wall. Top row: Milligan’s trichrome, collagen stained in green. Bottom row: Orcein, elastin stained in dark brown. Scale bar = 200 μm. Data for the WS group were modified from a previous study^13^.

Milligan’s trichrome staining was used to quantify total collagen in the neointima as a further indicator of neointimal maturity. Collagen content within the neointima of HS grafts increased rapidly from week 6 post-implantation and continued to increase through to 24 weeks. Whereas collagen content in the neointima of WS grafts remained significantly lower from week 6. The collagen content in HS grafts was 166 % greater than WS grafts at 6 weeks post-implantation (46.1 ± 6.6 vs 17.3 ± 7.6) (Fig. 6A).

Increased elastin content within the graft is a sign of positive graft remodelling as elastin is an important component of native arteries which imparts favourable mechanical properties to the synthetic graft. Elastin was quantified with Orcein staining, where elastin is stained in brown/black. At early timepoints, HS grafts demonstrated increased elastin in the neointima relative to WS graft, the most striking difference being at week 6 where elastin in HS grafts was 70 % higher than WS grafts (27.8 ± 2.8 vs 15.8 ± 2.8). Variability in the presence of elastin in the sections meant that these differences did not reach statistical significance. Quantification of each segment of the graft of HS grafts showed elastin preferentially depositing adjacent to the anastomoses (Fig. 6B). Representative images of HS grafts showed most elastin on the luminal side of the neointima, becoming more diffuse towards the graft wall (Fig. 6C, bottom row). A direct comparison of collagen and elastin stained images for WS and HS grafts can be seen in Supplementary Fig. 7.

#### Inflammation

**Figure 7:**
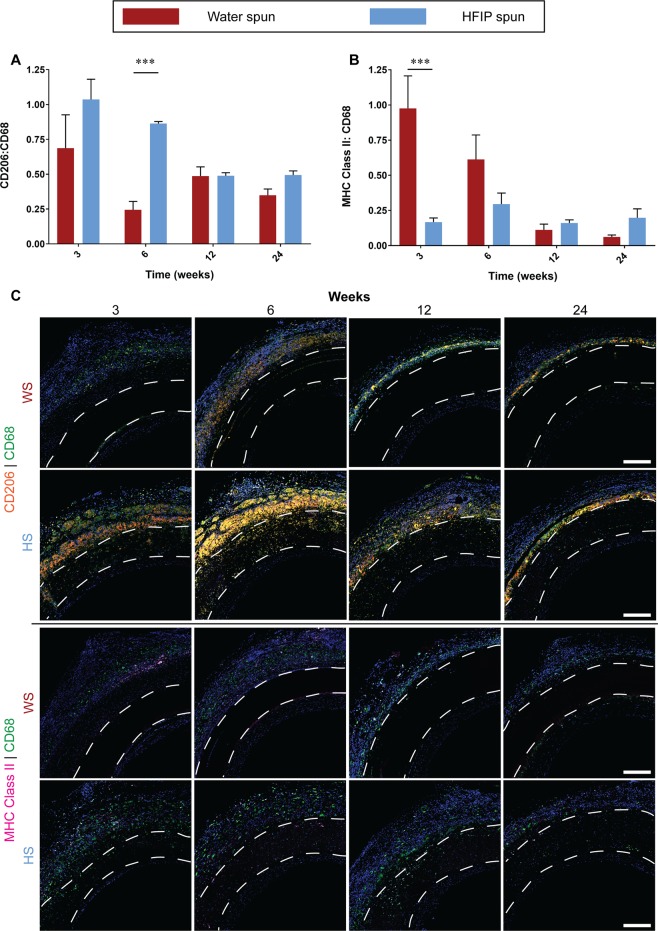
Macrophage response to electrospun silk grafts. Data expressed as mean ± SEM, n = 4 animals/timepoint. Statistical analysis comparing these averages was by two-way ANOVA, using Šídák multiple comparison test, with *p < 0.05, **p < 0.01, ***p < 0.001. (**A**) Quantification of M2 polarisation represented as a ratio of CD206:CD68. (**B**) Quantification of M1 polarisation represented as a ratio of MHC Class II:CD68. (**C**) Representative images of cross sections at the proximal end of HS silk vascular grafts, white dotted lines indicate the graft wall. Upper two rows: CD206 stained in orange, CD68 stained in green and nuclei stained in blue. Lower two rows: MHC Class II stained in purple, CD68 stained in green and nuclei stained in blue. Scale bar = 200 μm.

Localised chronic inflammation is a major driver of neointimal hyperplasia. Macrophages are a major contributor to chronic inflammation. We recently demonstrated a link between macrophage phenotype, local inflammatory environment and subsequent graft performance, with CD206^+^ M2 macrophages associated with improved performance^14^. Total macrophages were stained using the CD68 marker and their individual M1 (pro-inflammatory) and M2 (anti-inflammatory) phenotypes were differentiated using MHC Class II and CD206, respectively. HS grafts showed a non-significant increase in the number of M2 macrophages compared to WS grafts at the earliest timepoint of 3 weeks (p = 0.0591). This difference became significant at 6 weeks, where HS grafts showed a 234 % increase in M2 macrophages relative to WS grafts (0.863 ± 0.03 vs 0.258 ± 0.06) (Fig. 7A). Representative images showed that majority of the macrophages accumulated at the adventitial wall of the graft with minimal presence of macrophages in the neointima (Fig. 7C, top 2 rows).

In contrast, analysis of M1 macrophages showed the opposite trends. WS grafts had 476 % higher M1 macrophage accumulation at week 3 (0.98 ± 0.23 vs 0.17 ± 0.03). This trend continued through to week 6 post implantation where WS grafts were higher but statistical significance was lost (Fig. 7B). Timepoints of 12 and 24 weeks showed no difference between the two groups. Similar to M2 macrophages, the majority of the macrophages were found on the adventitial wall of the vascular grafts (Fig. 7C, bottom 2 rows).

## Discussion

Current synthetic vascular graft materials uniformly fail in low diameter (<6 mm) applications such as coronary artery and lower limb bypass^2^. There is a large unmet need for a cost effective, acellular synthetic vascular graft with scalable manufacturing as an off-the-shelf solution. Silk fibroin is a natural protein, FDA-approved for suture and dermal applications, and can be cheaply manufactured on a large-scale^15,16^. Advances in silk purification have significantly improved silk purity and made production of mechanically-robust conduits feasible for the first time^5^. In our previous studies, we demonstrated a new synthetic silk fibroin graft that has tailored mechanical properties and significantly enhanced survival and endothelialisation compared to ePTFE over 6 months as a rat aortic replacement^13^. In this study, we manufactured silk vascular grafts with significantly increased fibre diameter and porosity with the aim of enhancing cell infiltration and remodelling. Careful comparison of these two silk graft iterations allowed us to assess the effect of physical parameters on the biological response in rats.

Using electrospinning we generated two silk grafts with significantly different physical properties. The difference in parameters between HS and WS include the solvent, concentration of electrospinning solution and flow rate. The most prominent physical changes were seen in fibre thickness, porosity and cell infiltration *in vitro*. Previous studies using electrospun silk have dominantly used formic acid or water as solvents. While the production of polymer nanofibers from water solution is considered as environmentally advantageous, up-scalable and versatile^17,18^, previous work has reported a very narrow window of fiber thickness^18,19^. Consistent with this, our results showed that electrospinning from water resulted in fibers in the low nanometer ranges, largely unaffected by modulation of electrospinning flow rate or solution viscosity. Hexafluoroisopropanol (HFIP) is a common electrospinning solvent previously demonstrated for both natural and synthetic polymers, including tropoelastin^20^, collagen^21^ and polycaprolactone^22,23^. To our knowledge, HFIP has not been previously used to electrospin silk, perhaps because of concerns around denaturing the protein^24^. Electrospinning silk from HFIP allows a significant increase in silk concentration (and viscosity), providing an enhanced control over fiber diameter thus increasing the range of possible diameters into the micron range. Consequently, material porosity is increased, with higher concentrations of silk (15%) yielding the thickest fibers and largest distribution. Changes in mechanical properties in this study showed similar trends to previous studies where larger fibre diameters increased stiffness and burst pressure. For example, in electrospun polycaprolactone, thicker fibres corresponded with increased stiffness and tensile strength^25^. In this study, the increased strength could also stem from an increased concentration of silk between the iterations. Modifying physical graft properties is expected to lead to differences in physiological response, however, direct comparisons *in vivo* of different parameters are lacking.

In a rat abdominal aorta grafting model, we observed significantly improved graft performance in HS relative to WS silk as measured by key markers of efficacy. Re-endothelialisation is perhaps most critical; rapidly establishing a complete endothelium on the surface of the lumen is associated with reduced thrombogenicity and improved long-term patency^26,27^. Previous studies have examined the physical properties of biomaterials and the corresponding interactions with endothelial cells. *In vitro* assessment of scaffolds with fibre thickness of 2 µm had the fastest migration velocity in a study that compared fibre diameters between 0.5 µm to 10 µm^28^. In this study, we found that *in vivo* endothelialisation was significantly improved on HS compared to WS silk, enhanced ~ 150% at 3 weeks. This is consistent with similar studies where ePTFE and polyurethane with a range of porosity was assessed *in vivo*^29,30^. PU grafts with the largest pore size elicited the highest rate of endothelialisation due to infiltration of perivascular tissue to support endothelial growth^30^. Studies investigating transmural endothelialisation, a process in which capillary ingrowth from the perivascular tissue contributes to the endothelium at the lumen, also showed that graft porosity is an important factor^31,32^. The observed rapid endothelialisation in HS silk could also be attributed to increased endothelial progenitor contributions. Endogenous stem cells and progenitor cell homing to implanted biomaterials have been shown to play significant roles in vascular graft remodelling^33^. While most studies aiming to increase endothelial progenitor cell contribution have focused on chemotactic functionalization^34,35^, one study has proposed that the alignment of electrospun fibres are equally important in the context of EC migration^36^. The data shown here suggests that changes to the physical properties of HS silk, independent of any changes to chemotactic cues drove a greater proportion of CD34^+^ endothelial progenitor cells relative to WS silk.

Neointimal hyperplasia is another major consideration for long-term vascular graft survival. The comparison between WS and HS silk in this study suggest unique temporal neointimal hyperplasia development profiles. WS silk showed a dramatic increase in neointimal area from 3 weeks to 6 weeks followed by contraction to a stable level. In contrast, HS silk showed only a gradual increase from 3 weeks through to 24 weeks, stabilising much more rapidly than WS silk. The phenomenon of neointimal regression has been described in previous studies with stenting models and venous grafts^37,38^. Possible drivers of neointimal regression suggested in these studies include high shear stress which regulate SMC proliferation and apoptosis at the neointima. While the exact molecular mechanisms of neointimal regression remain unclear and was not a subject of the study, our data on SMC and proliferating cells within the neointima showed that HS silk elicited a significantly earlier contractile SMC phenotype switch compared to WS. This contributed to the steady neointimal hyperplasia progression and lower stabilisation levels of neointimal hyperplasia at later timepoints. A previous study comparing the neointimal hyperplasia response of grafts with different porosity showed that SMC in the non-porous graft underwent transdifferentiation to cause calcification whereas SMC in the porous grafts maintained expression of contractile SMC markers^39^. This observation was reaffirmed when highly porous PCL graft showed no calcification after one year in a rat abdominal aorta grafting model^40^. ECM production of cells populating the neointima also reflects the difference in the SMC phenotypical switch. Collagen in the neointima of HS silk increased and stabilised at a much higher percentage of the neointima compared to WS silk. Similarly, increase in elastin content in the neointima of HS silk at 6 weeks coincided with significantly higher endothelialisation. Elastin regulates SMC phenotype, with higher elastin concentrations leading to preserved contractile phenotype^41,42^. Another reason for the difference in temporal neointimal hyperplasia response could be due to the greater endothelialisation observed in HS silk at earlier timepoints. Endothelial cells are known to have signalling pathways which inhibit neointimal hyperplasia^43^. ECs secrete signalling molecules such as NO, which inhibit cell proliferation^44^. Since we observe a faster establishment of endothelium in the HS silk, it is reasonable to expect the corresponding changes in SMC phenotype to favour a stabilisation of neointima development. The rate of graft degradation is also expected to play a role in these remodelling and healing responses. We observed only modest degradation in this time course and relied on histology for an initial assessment. Further studies will explore changes to mechanical properties and fibre integrity at explant to elucidate this contribution.

Given the striking difference seen between the two groups, we made preliminary investigations into the underlying causes of these functional benefits. Previous studies have shown that manipulation of the surface geometry and morphology of a biomaterial can elicit vastly different biological responses^45^. *In vitro* experiments with bone marrow derived macrophages showed that thicker fibres with larger pores were able to modulate macrophages to the reparative M2 phenotype^46^. This was further explored *in vivo* with electrospun polycaprolactone vascular grafts, where fibre diameters up to 6 µm were implanted and assessed^25^. The authors showed a sustained expression of the M2 marker, CD206, in the large diameter fibre graft which translated to substantial remodelling of the graft to resemble a native artery. In this study, we were able to directly compare the effects of the fibre diameter of electrospun silk vascular grafts *in vivo*. More recently, studies have shown that formation of neutrophil extracellular traps (NETs) are also influenced by the fibre diameter, with larger fibres eliciting less NETs formation relative to low diameter fibres^47^. These early inflammatory responses may have long term effects on vascular graft remodelling and further assessment at acute timepoints would help elucidate these contributions.

## Conclusion

Utilising the advantages of electrospinning, we made a new iteration of a previously developed silk graft with significantly increased fibre diameter and porosity. The resulting silk graft showed striking functional benefits *in vivo* assessment in a rat abdominal aorta grafting model for up to 24 weeks. We observed significantly faster endothelialisation and overall lower neointimal area when compared to the less porous graft which coincided with an early SMC phenotypical switch to contractile phenotype. Here, we suggest that these differences in vascular remodelling could be due in part to a difference in the macrophage response. The high porosity graft elicited a higher anti-inflammatory M2 macrophage response at early timepoints, while having lower pro-inflammatory M1 macrophages. Together, the results of this study justify further evaluation of silk fibroin conduits in pre-clinical grafting models.

## Methods

### Purification of silk fibroin

*Bombyx mori* silk cocoons were purchased from Tajima Shoji Co., Ltd., Yokohama, Japan and purified as previously described^5^. Briefly, batches of the cleaned silk cocoons (5 g) were boiled for 30 minutes in sodium carbonate (0.02 M, 2 L) to remove sericin, the glue-like protein component of the silk fibre. The resulting silk fibres were thoroughly washed with water and left to dry overnight and stored at room temperature until further use. Silk fibres were dissolved in freshly prepared lithium bromide solution (9.3 M) at 25 % (w/v) for 3 hours at 60 °C. The dissolved silk was then dialysed using SnakeSkin™ dialysis tubing (3.5 kDa molecular weight cut off; Thermo Fisher) in milliQ water to remove traces of lithium bromide, with a total of 6 changes in dialysis solution over 3 days. The silk solution was collected from the dialysis tubing and centrifuged (2 × 8700 rpm, 20 min, 4 °C) The concentration (w/w) of the silk solution was determined by drying a known mass of the aqueous silk solution and weighing the remaining solid silk. Aqueous silk solution was stored at 4 °C for use within a one-month interval. Alternatively, silk solutions were lyophilised and stored at 4 °C with desiccant beads until further use.

### Viscosity measurements

Viscosity of all electrospun silk solutions was measured on a Malvern Kinexus Ultra+ (Malvern Instruments Ltd.) as previously reported^48^. We used a flat 25 mm parallel plate upper geometry with a gap of 1mm and 0.3 mm for the aqueous silk and HFIP silk solutions, respectively. A shear rate ramp of 0.1 to 100s-1 was applied over 2 minutes and the viscosity measured. The results displayed are for the shear rate to which the silk solutions (aqueous or in HFIP) are subject to during the electrospinning process, assuming Newtonian fluid dynamics. Results are plotted as viscosity (η) in Pa.s.

### Electrospinning

Electrospinning of WS silk was performed from aqueous silk, with the addition of PEO to increase silk viscosity^49^. Different silk/PEO ratios of 2 mL silk:1g PEO, 2 mL silk:2g PEO, 2 mL silk:3g PEO and 2 mL silk:4g PEO were tested. Spinning was performed at flow rates of 0.7 mL/hr, 1.5 mL/hr and 2.0 mL/hr at a voltage of up to 20 kV. Electrospinning of HS silk was performed from lyophilised silk, dissolved in hexafluoroisopropanol (HFIP) at 5%, 10%, 15% and 20% (w/v). Flow rates of 1 mL/hr, 1.5 mL/hr, 2.0 mL/hr and 2.5 mL/hr were tested. All electrospinning was performed at 18 cm, with mats and grafts electrospun on 20 mm and 1.5 mm diameter mandrels, respectively, rotating at 500 rpm. To stabilise electrospun silk mats and tubes under aqueous conditions, water annealing was performed in a humidified chamber (Wheaton) under vacuum at 4, 24, 37 or 55 °C overnight to induce beta-sheet formation^5^. HFIP spun scaffolds were washed in 5% sodium lauryl sulfate, to remove residual HFIP, and washed three times with and stored in sterile PBS. Electrospun silk grafts were disinfected in ethanol (70%), air dried then exposed to UV light for 30 min. Samples were stored in air until the time of surgery.

### *In vitro* cell infiltration

Scaffolds with a diameter of 6 mm were cut using a circular punch biopsy and adhered to the surface of a 96-well plate. Scaffolds were rehydrated in hCAEC media (Cell Applications Inc.) overnight, prior to cell experiments. Human Coronary Artery Endothelial Cells (Cell Applications Inc.) at passages 3–5 were seeded on silk scaffolds a density of 25000 cells/cm^2^ and left for 9 days, with daily media replacement and re-seeding at day 3. Cells were fixed and embedded in resin (JB-4, Polysciences Inc.) and 3 μm sections were cut perpendicular to the graft and stained with Mayer’s Hematoxylin & Eosin. Quantification was performed by manual counting from 6 scaffold sections, for 3 replicate samples. The distance of the cell nucleus to the scaffold surface was measured and is represented in μm of cell migration into the scaffold.

### Mechanical testing

Mechanical testing of the silk mats was performed on an Instron Tensile Machine (Instron 5543), as we previously published^13^. Electrospun silk mats were cut into strips (0.5 mm × 3.5 mm) and clamped into the Instron Tensile Machine. The length, width, and thickness of each sample were measured using digital calipers before each test. A constant pull of 3 mm/min was applied, whilst samples were submersed in PBS at 37 °C. The force was measured by a 50 N load cell and data is output in the form of N/s. The data was analysed and a stress/strain curve for each replicate mat was obtained. The elastic modulus was obtained from the linear region of the stress/strain curve accounting for material surface area. Ultimate tensile strength (UTS) is defined as the maximum force the material can withstand before breaking.

Suture pull-out studies have also been previously described^13^. Briefly, electrospun silk grafts with a 1.5 mm internal diameter were cut into 1.25 cm sections. Two sections were sutured together with 8 interrupted 9–0 sutures (Johnson & Johnson), joining the two cut surfaces. Suture pull out was performed on an Instron Tensile Machine (Instron 5543) with a constant pull rate of 3 mm/min and the force measured by a 50 N load cell, whilst submerged in PBS at 37 °C. The suture pull-out is an expression of the highest force at which the two halves were still held together by the 8 interrupted sutures.

Burst pressure measurements were performed as previously described by attaching a 2.5 cm section of electrospun silk grafts, with an internal diameter of 1.5 mm, to an 18 G cannula connected to a pressure transducer (Cambridge Electronic Design) connected to a bio-amplifier (BMA-931, Cambridge Electronic Design)^50^. Pressure in the graft was increased by pumping (Harvard Apparatus) vaseline into the graft at a rate of 0.1 mL/min. The pressure was recorded as a continuous wave-form using Spike2 acquisition and analysis software (Version 8.03; Cambridge Electronic Design) and digitized at 250 Hz. Burst pressure is an expression of the highest pressure before the graft ruptured.

### Rat abdominal aorta grafting

The study was conducted as we have previously described^13^. Approval was obtained from the Sydney Local Health District Animal Welfare Committee (protocol number 2014/028). Experiments were conducted in accordance with the Australian Code of Practice for the Care and Use of Animals for Scientific Purpose.

Rats (Sprague Dawley, male, 8 weeks) were purchased from Laboratory Animal Service (NSW, Australia). Rats were given a single intramuscular injection of ketamine (75 mg/kg) and medetomidine (0.5 mg/kg) to induce anaesthesia. The abdomen was shaved and midline laparotomy incision was performed. The infrarenal abdominal aorta was exposed and clamped proximally; just below the renal arteries, and distally; just above the aortoiliac bifurcation. The abdominal aorta was resected and a 0.8 cm section of silk graft was implanted with 8 interrupted 9–0 silk sutures at each anastomosis. After re-establishment of blood flow, the abdominal cavity and overlying skin were sutured closed. At the end of surgery, rats were given atipamazole (in equal volume to medetomidine; intramuscularly) to reverse the anaesthesia. For pain management, Temgesic (Buprenorphine 2 mg/kg bodyweight) was provided once a day for 24–48 hr. Animals were kept on normal food and water ad libitum for the duration of the study and no ongoing anticoagulants were given. Grafts were explanted at 3, 6, 12 and 24 weeks (n = 5 per time point) for scanning electron microscopy and histological analysis.

### Histology

Explanted grafts were fixed overnight in paraformaldehyde (4 %) at room temperature. Samples were dehydrated through an ethanol gradient and embedded in paraffin and sectioned at 5 µm transversely from proximal anastomosis to distal anastomosis^51^. For staining, slides from 6 equidistant points throughout the graft were deparaffinized and immediately stained with Haematoxylin and Eosin, Milligan’s trichrome for collagen deposition, orcein for detection of elastin^13^. For immunohistochemistry, slides were deparaffinized and stained with primary antibodies against vWF (1:500, A0082, Dako) and CD34 (1:250, ab81289, Abcam) for endothelial cells, smooth muscle α-actin (1:500, a5691, Sigma-Aldrich) for smooth muscle cells, PCNA (1:500, ab29, Abcam) for proliferative cells, CD68 (1:500, ab125212, Abcam) as a broad macrophage marker, CD206 (1:200, ab64693, Abcam) for M2 macrophages and MHC Class II (1:200, ab180779, Abcam) for M1 macrophages. Secondary antibody against rabbit (1:250, ab150080, Abcam and 1:250, ab150077, Abcam) were used to detect primary antibodies.

### Scanning electron microscopy

Explanted grafts were cut open longitudinally then fixed overnight in paraformaldehyde (4 %) at room temperature followed by glutaraldehyde (2.5 %) for one hour at room temperature, as previously described^51^. Samples were then post-fixed with osmium tetroxide (1 %) in phosphate buffer (0.1 M) and dehydrated through an ethanol gradient before drying using a critical point dryer (Leica Microsystems CPD300). Samples were gold sputter coated and imaged with a Zeiss Sigma VP FEG.

### Quantitative analysis

Fibre diameter and analysis of histological stains was quantified with ImageJ, as previously described^51^. Paraffin-embedded sections were selected from 6 points evenly distributed along the graft length. Endothelial coverage was quantified by measuring the circumference of vWF or CD34 positive staining along the lumen and expressed as a percentage of the total luminal circumference. Neointimal area was represented as a percentage defined as the neointimal area divided by total lumen area within the inner graft wall. SMC content was quantified by measuring the area of SM α-actin or PCNA positive staining using a constant threshold intensity and expressed as a percentage of the neointima area. Similarly, collagen and elastin were quantified and expressed as a percentage of the neointimal area. Macrophage response to the grafts were quantified by measuring positive staining for CD206, CD68 and MHC Class II, using a constant threshold intensity and expressed as a percentage of the total cross section of the graft. M1 and M2 macrophages percentages were calculated by positive CD206 or MHC Class II staining, respectively, normalised to CD68 staining in identical regions.

### Statistical analysis

Data are expressed as mean ± standard error of the mean (SEM) and indicated in figures as *p < 0.05, **p < 0.01, ***p < 0.001. Data was compared using two-way ANOVA analysis and Šídák multiple comparison test of the means of WS and HS graft across all time points with GraphPad Prism version 6 (GraphPad Software).

## Supplementary information

Supplementary Figures

## Data Availability

All data generated or analysed during this study are included in this published article and supplemental material.
