# Supplementary material for: Altered processing enhances the efficacy of small-diameter silk fibroin vascular grafts
Source: Sci Rep. 2019 Nov 25;9:17461. doi: 10.1038/s41598-019-53972-y (PMC6877724; doi:10.1038/s41598-019-53972-y)
Supplement: Supplementary file 1 — Supplementary Figures [file 41598_2019_53972_MOESM1_ESM.pdf]

Altered processing enhances the efficacy of small-diameter silk fibroin vascular grafts.

Alex H.P. Chan[1,2#], *Elysse C. Filipe*[3,4#], *Richard P. Tan*[1,2], *Miguel Santos*[1,2], *Nianji Yang*[1], *Juichien Hung*[1], *Jieyao Feng*[1], *Sidra Nazir*[1], *Alexander J. Benn*[1], *Martin K.C. Ng*[2,5], *Jelena Rnjak-Kovacina*[6*], *Steven G. Wise*[1,2,7,8*]

[1] *The Heart Research Institute, 7 Eliza Street, Newtown, Sydney, NSW 2042, Australia.*

[2] *Sydney Medical School, University of Sydney, NSW 2006, Australia*

[3] *Garvan Institute of Medical Research & The Kinghorn Cancer Center, Sydney, NSW 2010, Australia*

[4] *St Vincent's Clinical School, Faculty of Medicine, UNSW Sydney, NSW 2010, Australia*

[5] *Department of Cardiology, Royal Prince Alfred Hospital, Camperdown, NSW 2050, Australia*

[6] *Graduate School of Biomedical Engineering, UNSW Sydney, Sydney, NSW 2052, Australia*

[7] *School of Medical Sciences, Dept of Physiology, University of Sydney, NSW 2006, Australia*

[8] *Charles Perkins Centre, University of Sydney, NSW 2006, Australia*

Equal 1st Authors

* To whom correspondence should be addressed

Address for Correspondence

‖ Dr. Steven G. Wise

The Heart Research institute, 7 Eliza Street Newtown, NSW 2006 Australia

Email: steven.wise@sydney.edu.au Phone: +61286279458

Or

‖ Dr. Jelena Rnjak-Kovacina

Graduate School of Biomedical Engineering, University of New South Wales, NSW 2052, Australia

Email: j.rnjak-kovacina@unsw.edu.au Phone: +61293853920

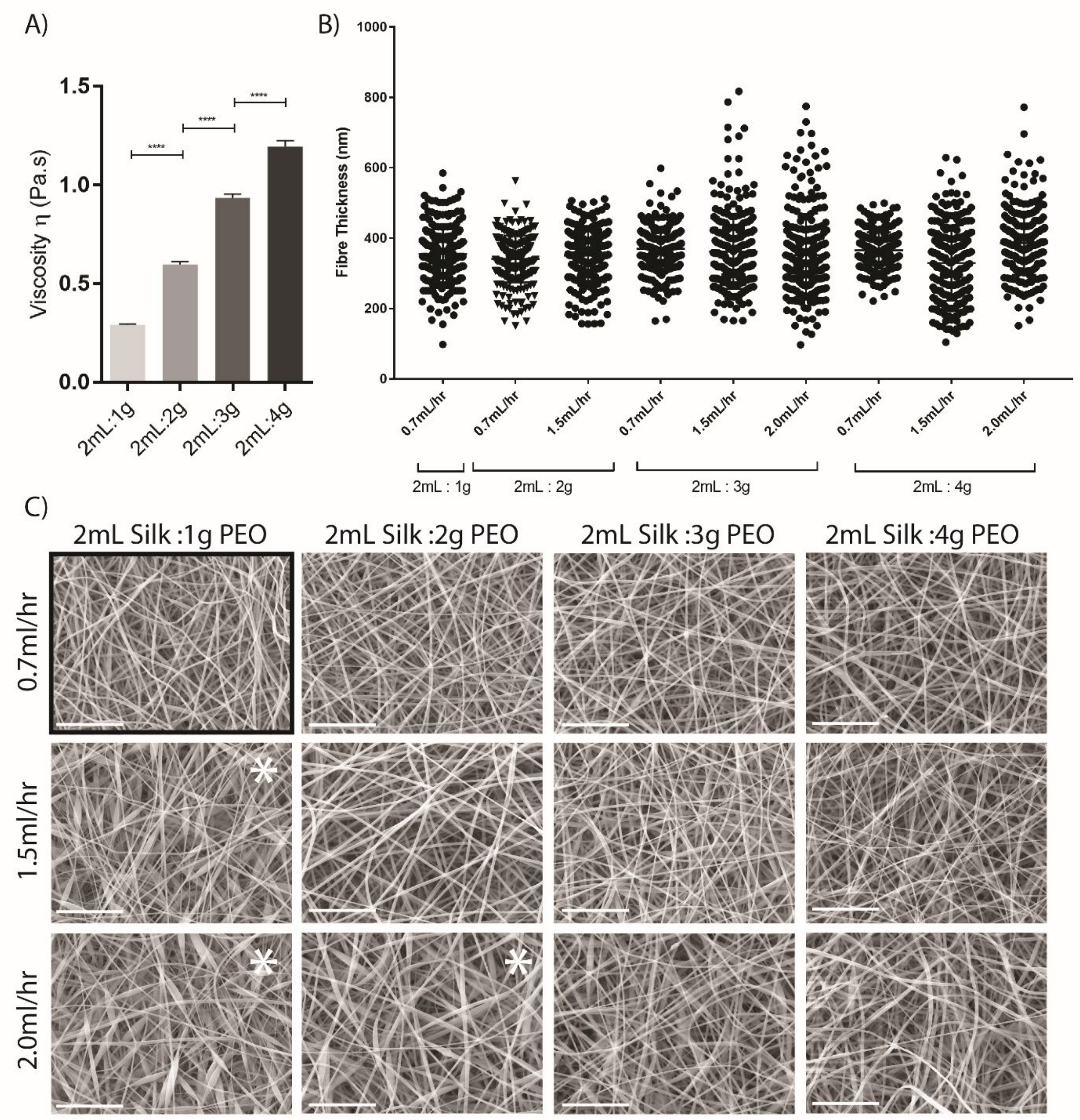

Supplementary Figure 1. Water electrospun Silk. A) Rheometry measurements of the viscosity of various silk/PEO samples prior to electrospinning. Data is expressed as mean ± SEM, n=6. B) Fibre thickness analysis plot, each dot representing one fibre measurement. C) SEM images of the various silk/PEO ratios at 0.7mL/hr, 1.0mL/hr and 1.5mL/hr flow rate. White asterisks indicate samples with beaded morphology (indicated with white arrow head). Scale bar = 10µm. **** = p<0.0001. Black box representing sample which was chosen for further studies.

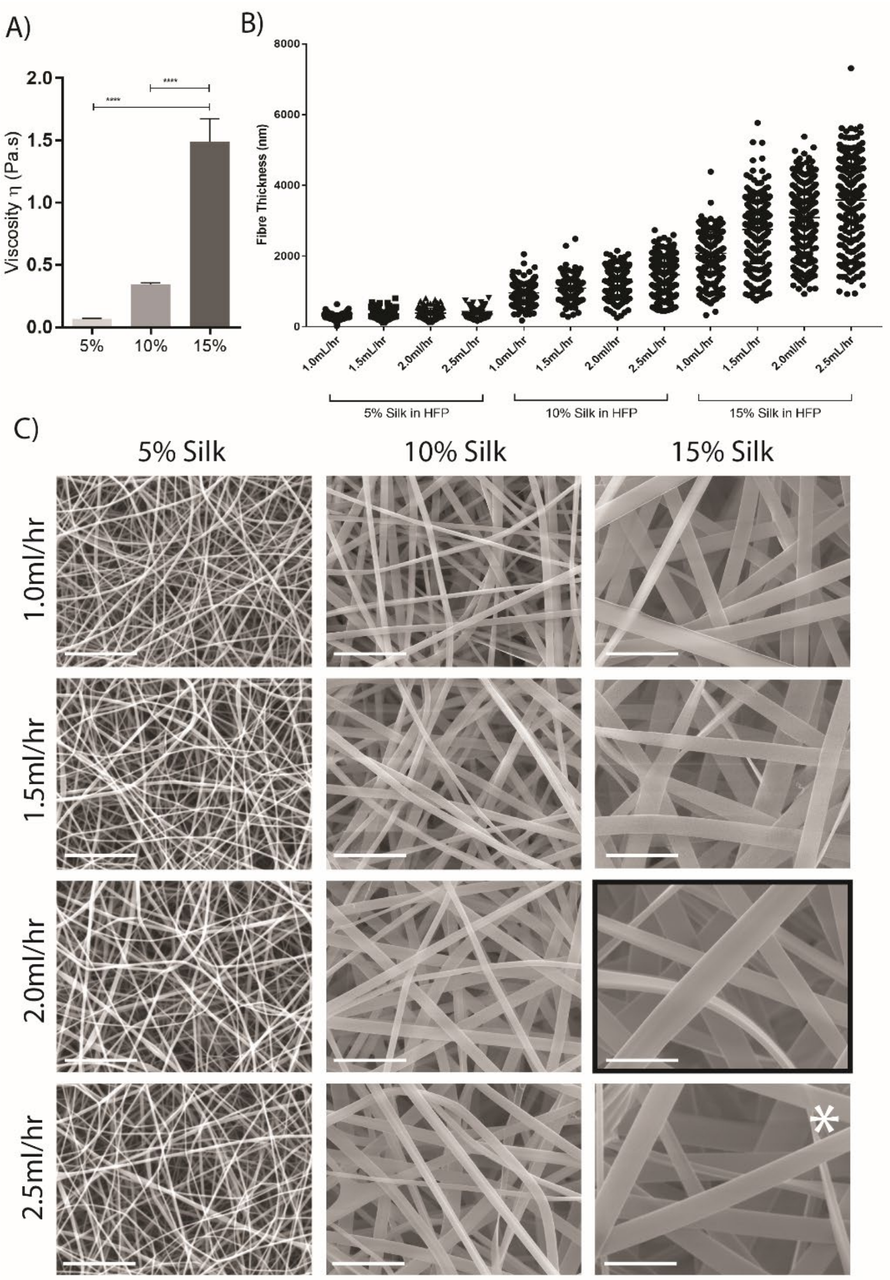

Supplementary Figure 2. HFIP electrospun Silk. A) Rheometry measurements of the viscosity of various HFIP silk samples prior to electrospinning. Data is expressed as mean ± SEM, n=6. B) Fibre thickness analysis plot, each dot representing one fibre measurement. C) SEM images of the various silk concentrations of 5%, 10% and 15% at 1.0mL/hr, 1.5mL/hr, 2.0mL/hr and 2.5mL/hr flow rate. White asterisk indicated sample which were difficult to electrospin. Scale bar = 10µm. **** = p<0.0001. Black box representing sample which was chosen for further studies.

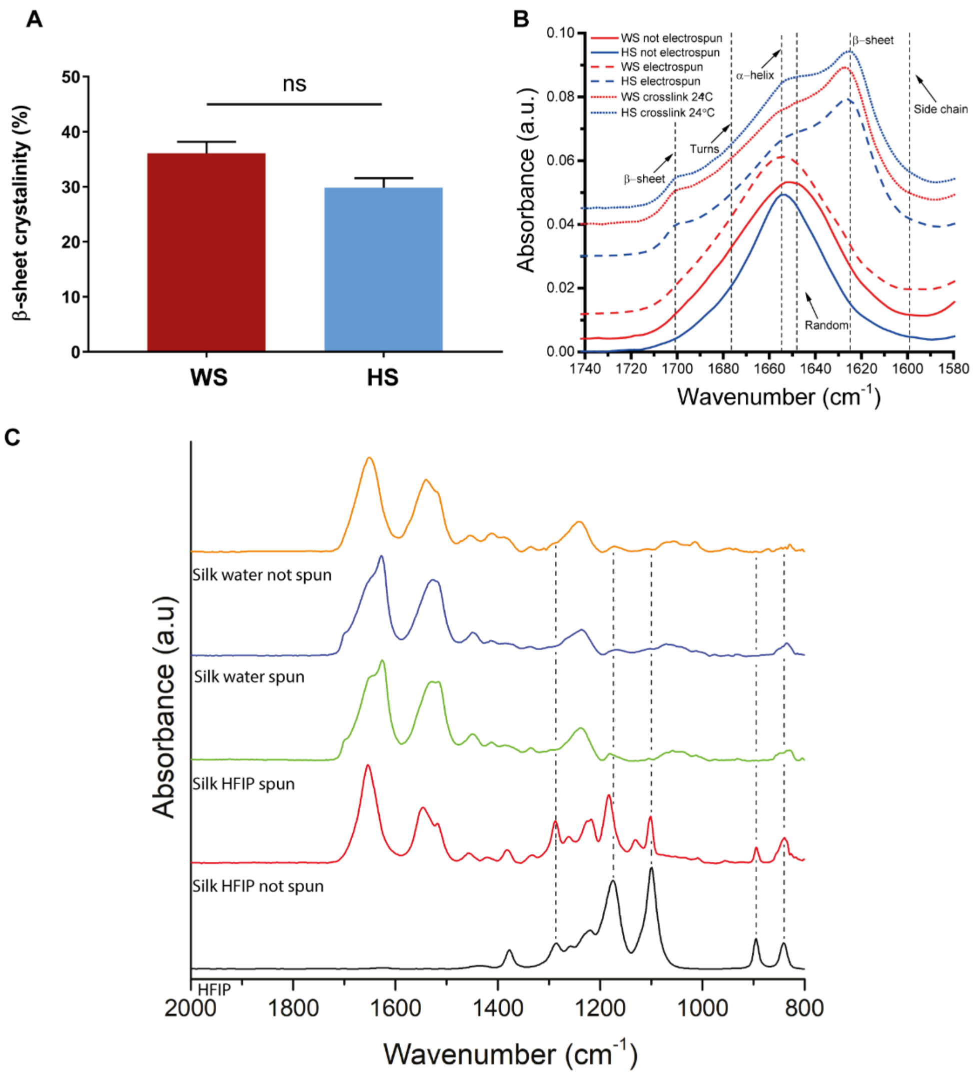

Supplementary Figure 3. Secondary structure of electrospun silk. A: Quantification of β-sheet crystallinity using FTIR. B: FTIR spectral window showing amide I bands for silk samples before electrospinning, after being electrospun and crosslinked at room temp. An increase in β-sheet crystallinity was observed immediately after electrospinning of HS silk and only after crosslinking for WS formulations as showed by a shift of the amide I band from around 1650 cm^{-1} (α-helixes) to 1625 cm^{-1} (β-sheets). C: FTIR spectra shows characteristic HFIP bands on silk dissolved in HFIP before electrospinning. However, the same HFIP bands were not detected following electrospinning and washing of HS silk, suggesting there is minimal if any solvent residues left in this formulation. Spectra of WS before and after electrospinning are also shown for comparison as these formulations were not in contact with HFIP.

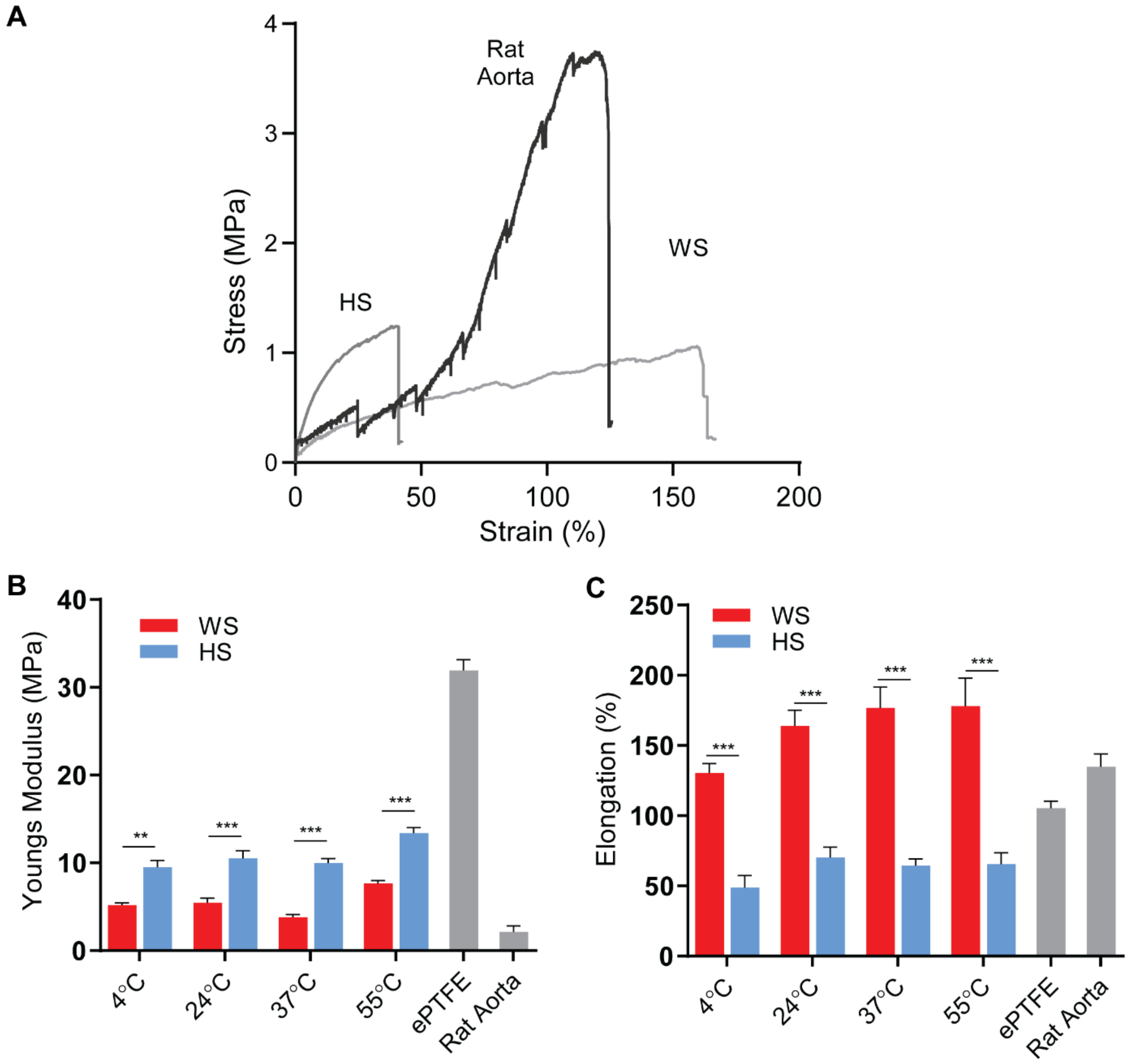

Supplementary Figure 4. A) Representative stress-strain curves for WS and HS materials, compared to rat aorta. B) Young's moduli and C) Percent elongation of WS and HS silk at different water annealing temperatures. Data is expressed as mean ± SEM, n=9-12 (n=2 for rat aorta). ** = $p<0.01$, *** = $p<0.001$.

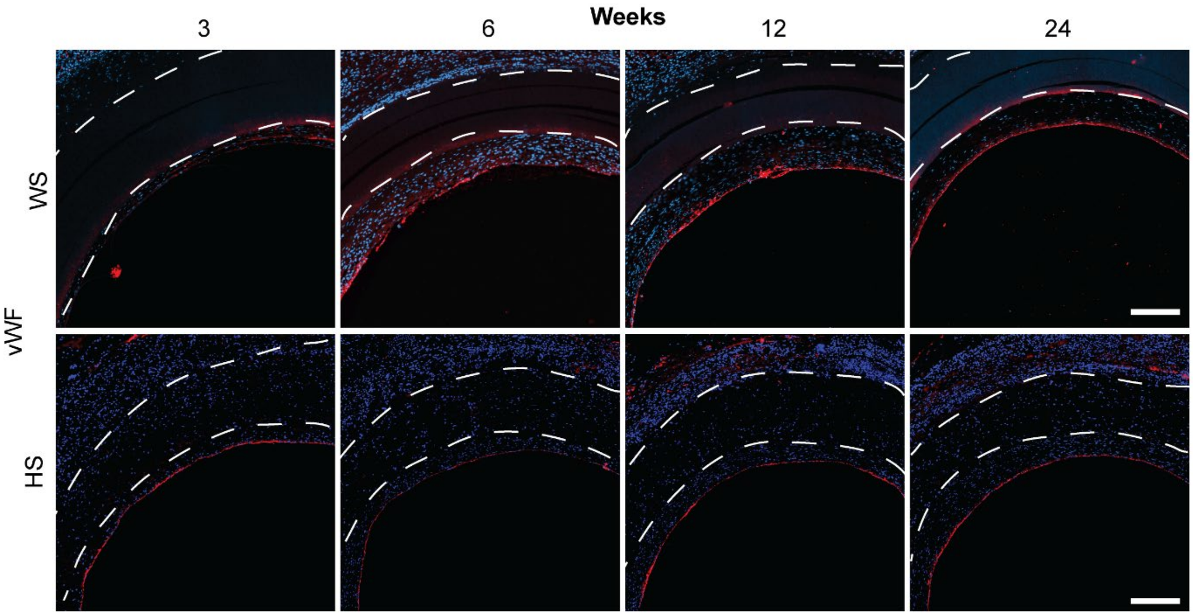

Supplementary Figure 5. Endothelialisation of silk vascular grafts. Representative images of cross sections at the mid graft of WS and HS silk vascular grafts, white dotted lines indicate the graft wall. vWF stained in red and nuclei in blue. Scale bar = 200 µm.

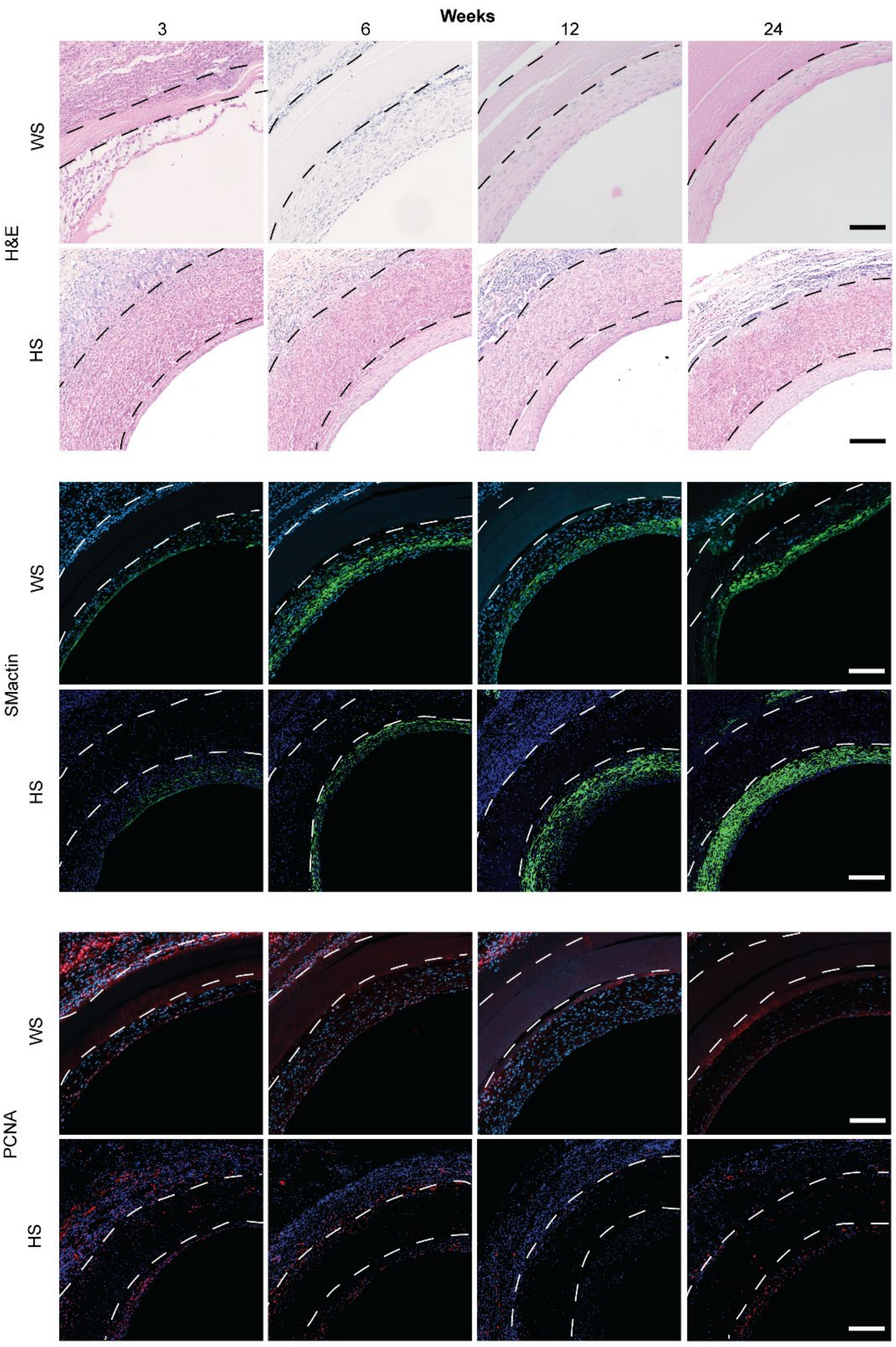

Supplementary Figure 6. Characterisation of neointimal hyperplasia development. Representative images of cross sections at the proximal end of WS and HS silk vascular grafts, black or white dotted lines indicate the graft wall. Top panel: Haematoxylin and eosin staining. Middle panel: SMactin stained in green and nuclei in blue. Bottom panel: PCNA stained in red and nuclei in blue. Scale bar = 200 μm.

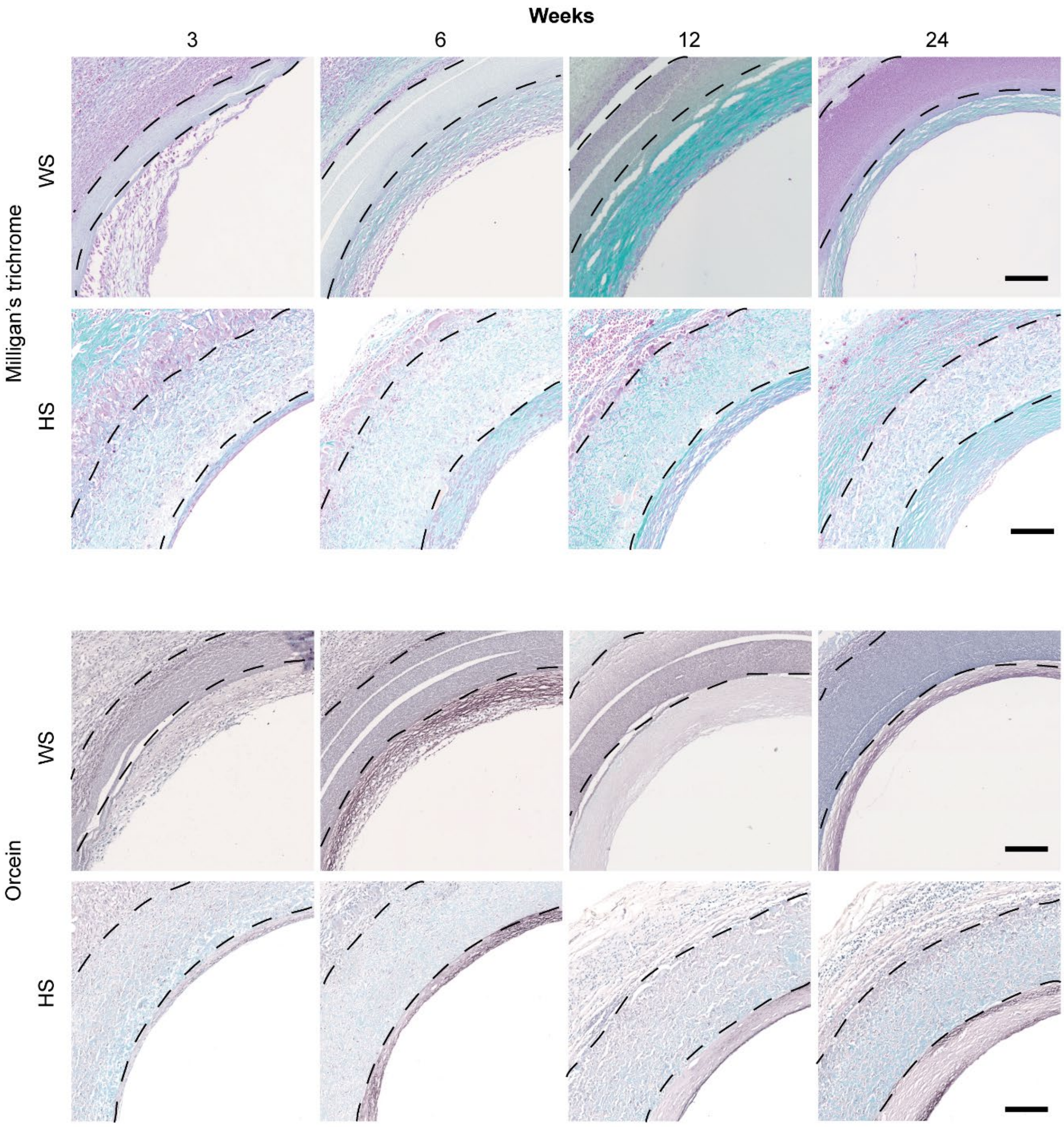

Supplementary Figure 7. Extracellular matrix deposition in the neointima. Representative images of cross sections at the proximal end of WS and HS silk vascular grafts, black dotted lines indicate the graft wall. Top panel: Milligan's trichrome, collagen stained in green. Bottom panel: Orcein, elastin stained in dark brown. Scale bar = 200 μm.
